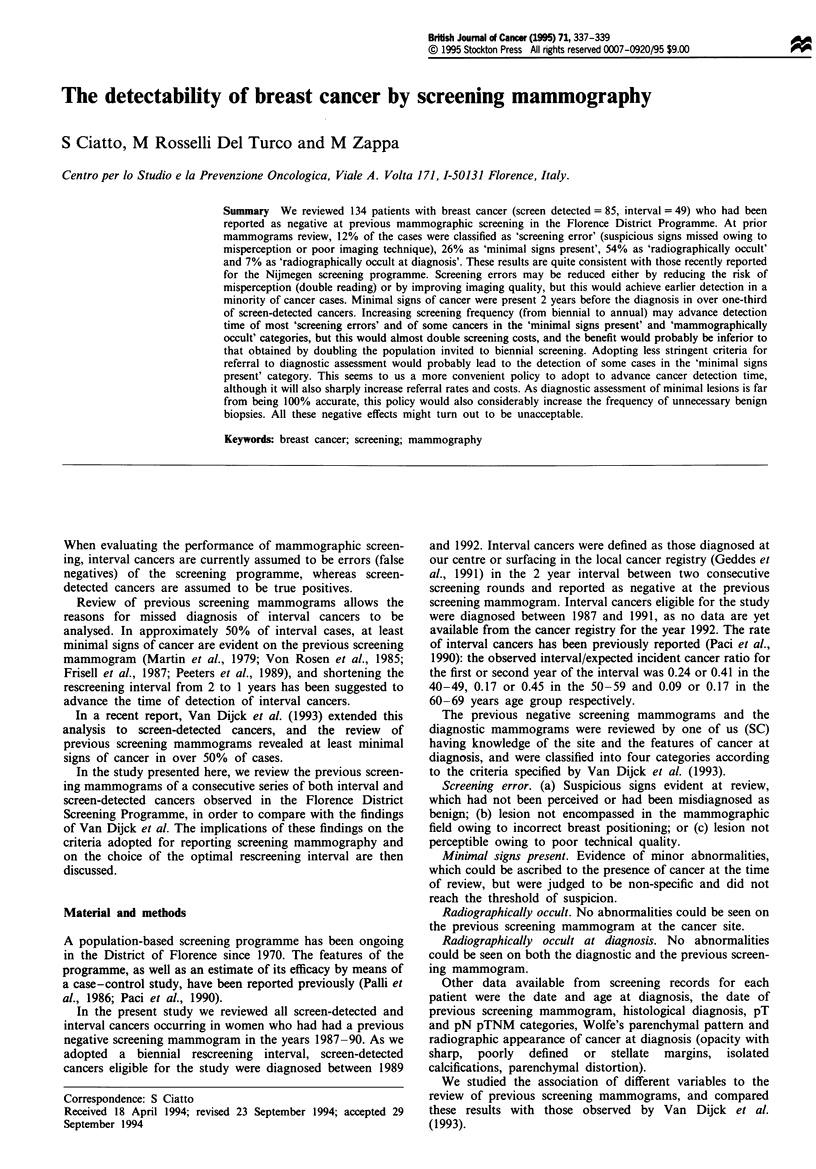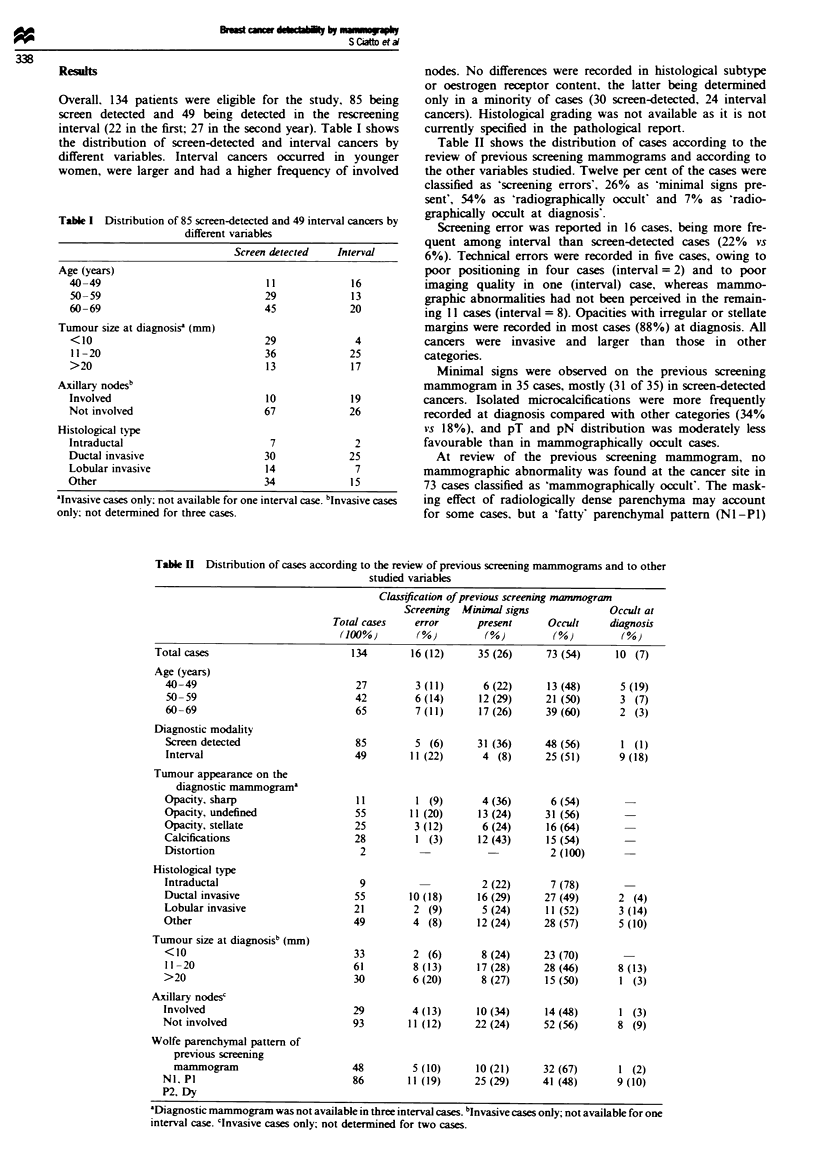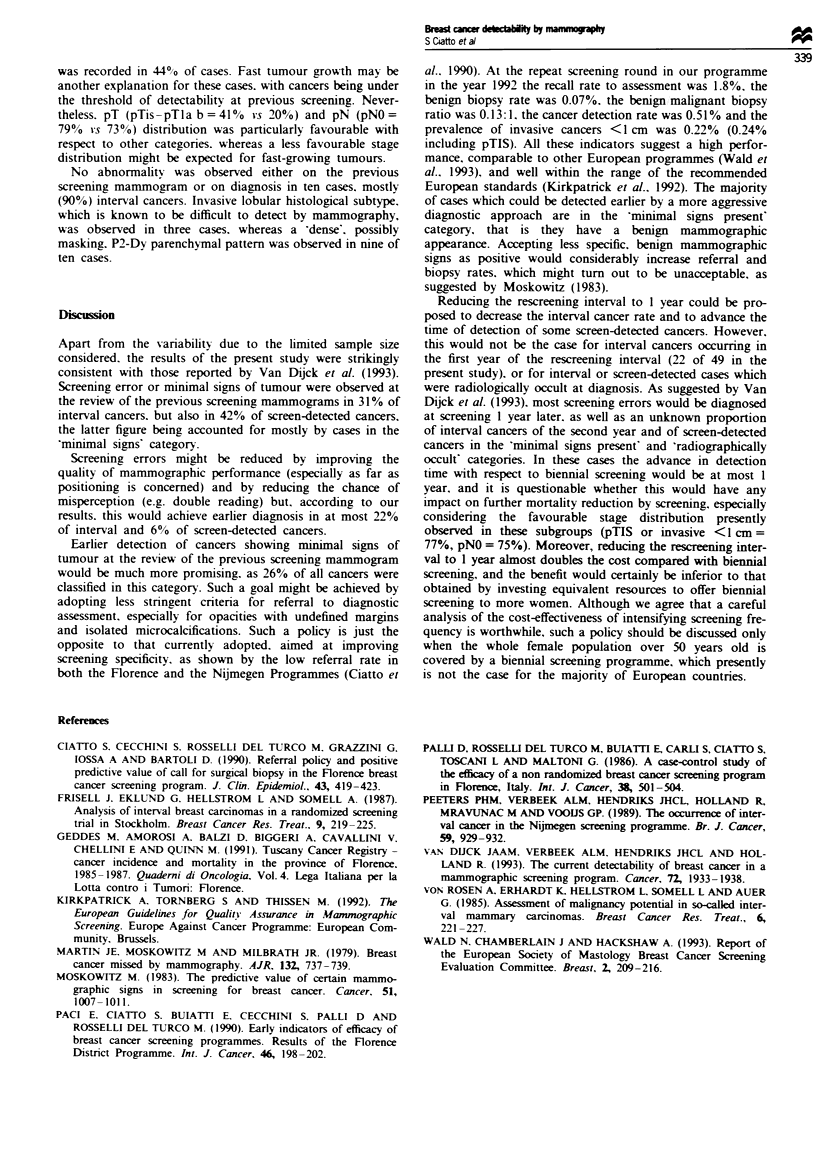# The detectability of breast cancer by screening mammography.

**DOI:** 10.1038/bjc.1995.67

**Published:** 1995-02

**Authors:** S. Ciatto, M. Rosselli Del Turco, M. Zappa

**Affiliations:** Centro per lo Studio e la Prevenzione Oncologica, Florence, Italy.

## Abstract

We reviewed 134 patients with breast cancer (screen detected = 85, interval = 49) who had been reported as negative at previous mammographic screening in the Florence District Programme. At prior mammograms review, 12% of the cases were classified as 'screening error' (suspicious signs missed owing to misperception or poor imaging technique), 26% as 'minimal signs present', 54% as 'radiographically occult' and 7% as 'radiographically occult at diagnosis'. These results are quite consistent with those recently reported for the Nijmegen screening programme. Screening errors may be reduced either by reducing the risk of misperception (double reading) or by improving imaging quality, but this would achieve earlier detection in a minority of cancer cases. Minimal signs of cancer were present 2 years before the diagnosis in over one-third of screen-detected cancers. Increasing screening frequency (from biennial to annual) may advance detection time of most 'screening errors' and of some cancers in the 'minimal signs present' and 'mammographically occult' categories, but this would almost double screening costs, and the benefit would probably be inferior to that obtained by doubling the population invited to biennial screening. Adopting less stringent criteria for referral to diagnostic assessment would probably lead to the detection of some cases in the 'minimal signs present' category. This seems to us a more convenient policy to adopt to advance cancer detection time, although it will also sharply increase referral rates and costs. As diagnostic assessment of minimal lesions is far from being 100% accurate, this policy would also considerably increase the frequency of unnecessary benign biopsies. All these negative effects might turn out to be unacceptable.


British Journal of Cancer (1995) 71, 337-339
© 1995 Stockton Press All rights reserved 0007-0920/95 $9.00

### The detectability of breast cancer by screening mammography

S Ciatto, M Rosselli Del Turco and M Zappa

*Centro per lo Studio e la Prevenzione Oncologica, Viale A. Volta 171, I-50131 Florence, Italy.*

**Summary** We reviewed 134 patients with breast cancer (screen detected = 85, interval = 49) who had been reported as negative at previous mammographic screening in the Florence District Programme. At prior mammograms review, 12% of the cases were classified as 'screening error' (suspicious signs missed owing to misperception or poor imaging technique), 26% as 'minimal signs present', 54% as 'radiographically occult' and 7% as 'radiographically occult at diagnosis'. These results are quite consistent with those recently reported for the Nijmegen screening programme. Screening errors may be reduced either by reducing the risk of misperception (double reading) or by improving imaging quality, but this would achieve earlier detection in a minority of cancer cases. Minimal signs of cancer were present 2 years before the diagnosis in over one-third of screen-detected cancers. Increasing screening frequency (from biennial to annual) may advance detection time of most 'screening errors' and of some cancers in the 'minimal signs present' and 'mammographically occult' categories, but this would almost double screening costs, and the benefit would probably be inferior to that obtained by doubling the population invited to biennial screening. Adopting less stringent criteria for referral to diagnostic assessment would probably lead to the detection of some cases in the 'minimal signs present' category. This seems to us a more convenient policy to adopt to advance cancer detection time, although it will also sharply increase referral rates and costs. As diagnostic assessment of minimal lesions is far from being 100% accurate, this policy would also considerably increase the frequency of unnecessary benign biopsies. All these negative effects might turn out to be unacceptable.

**Keywords:** breast cancer; screening; mammography

When evaluating the performance of mammographic screening, interval cancers are currently assumed to be errors (false negatives) of the screening programme, whereas screen-detected cancers are assumed to be true positives.

Review of previous screening mammograms allows the reasons for missed diagnosis of interval cancers to be analysed. In approximately 50% of interval cases, at least minimal signs of cancer are evident on the previous screening mammogram (Martin *et al.*, 1979; Von Rosen *et al.*, 1985; Frisell *et al.*, 1987; Peeters *et al.*, 1989), and shortening the rescreening interval from 2 to 1 years has been suggested to advance the time of detection of interval cancers.

In a recent report, Van Dijck *et al.* (1993) extended this analysis to screen-detected cancers, and the review of previous screening mammograms revealed at least minimal signs of cancer in over 50% of cases.

In the study presented here, we review the previous screening mammograms of a consecutive series of both interval and screen-detected cancers observed in the Florence District Screening Programme, in order to compare with the findings of Van Dijck *et al.* The implications of these findings on the criteria adopted for reporting screening mammography and on the choice of the optimal rescreening interval are then discussed.

### Material and methods

A population-based screening programme has been ongoing in the District of Florence since 1970. The features of the programme, as well as an estimate of its efficacy by means of a case–control study, have been reported previously (Palli *et al.*, 1986; Paci *et al.*, 1990).

In the present study we reviewed all screen-detected and interval cancers occurring in women who had had a previous negative screening mammogram in the years 1987–90. As we adopted a biennial rescreening interval, screen-detected cancers eligible for the study were diagnosed between 1989 and 1992. Interval cancers were defined as those diagnosed at our centre or surfacing in the local cancer registry (Geddes *et al.*, 1991) in the 2 year interval between two consecutive screening rounds and reported as negative at the previous screening mammogram. Interval cancers eligible for the study were diagnosed between 1987 and 1991, as no data are yet available from the cancer registry for the year 1992. The rate of interval cancers has been previously reported (Paci *et al.*, 1990): the observed interval/expected incident cancer ratio for the first or second year of the interval was 0.24 or 0.41 in the 40–49, 0.17 or 0.45 in the 50–59 and 0.09 or 0.17 in the 60–69 years age group respectively.

The previous negative screening mammograms and the diagnostic mammograms were reviewed by one of us (SC) having knowledge of the site and the features of cancer at diagnosis, and were classified into four categories according to the criteria specified by Van Dijck *et al.* (1993).

*Screening error.* (a) Suspicious signs evident at review, which had not been perceived or had been misdiagnosed as benign; (b) lesion not encompassed in the mammographic field owing to incorrect breast positioning; or (c) lesion not perceptible owing to poor technical quality.

*Minimal signs present.* Evidence of minor abnormalities, which could be ascribed to the presence of cancer at the time of review, but were judged to be non-specific and did not reach the threshold of suspicion.

*Radiographically occult.* No abnormalities could be seen on the previous screening mammogram at the cancer site.

*Radiographically occult at diagnosis.* No abnormalities could be seen on both the diagnostic and the previous screening mammogram.

Other data available from screening records for each patient were the date and age at diagnosis, the date of previous screening mammogram, histological diagnosis, pT and pN pTNM categories, Wolfe's parenchymal pattern and radiographic appearance of cancer at diagnosis (opacity with sharp, poorly defined or stellate margins, isolated calcifications, parenchymal distortion).

We studied the association of different variables to the review of previous screening mammograms, and compared these results with those observed by Van Dijck *et al.* (1993).

Correspondence: S Ciatto
Received 18 April 1994; revised 23 September 1994; accepted 29 September 1994

### Results

Overall, 134 patients were eligible for the study, 85 being screen detected and 49 being detected in the rescreening interval (22 in the first; 27 in the second year). Table I shows the distribution of screen-detected and interval cancers by different variables. Interval cancers occurred in younger women, were larger and had a higher frequency of involved nodes. No differences were recorded in histological subtype or oestrogen receptor content, the latter being determined only in a minority of cases (30 screen-detected, 24 interval cancers). Histological grading was not available as it is not currently specified in the pathological report.

**Table I** Distribution of 85 screen-detected and 49 interval cancers by different variables

| | *Screen detected* | *Interval* |
|---|---|---|
| Age (years) | | |
| 40–49 | 11 | 16 |
| 50–59 | 29 | 13 |
| 60–69 | 45 | 20 |
| Tumour size at diagnosis[a] (mm) | | |
| <10 | 29 | 4 |
| 11–20 | 36 | 25 |
| >20 | 13 | 17 |
| Axillary nodes[b] | | |
| Involved | 10 | 19 |
| Not involved | 67 | 26 |
| Histological type | | |
| Intraductal | 7 | 2 |
| Ductal invasive | 30 | 25 |
| Lobular invasive | 14 | 7 |
| Other | 34 | 15 |

[a]Invasive cases only; not available for one interval case. [b]Invasive cases only; not determined for three cases.

Table II shows the distribution of cases according to the review of previous screening mammograms and according to the other variables studied. Twelve per cent of the cases were classified as 'screening errors', 26% as 'minimal signs present', 54% as 'radiographically occult' and 7% as 'radiographically occult at diagnosis'.

Screening error was reported in 16 cases, being more frequent among interval than screen-detected cases (22% *vs* 6%). Technical errors were recorded in five cases, owing to poor positioning in four cases (interval = 2) and to poor imaging quality in one (interval) case, whereas mammographic abnormalities had not been perceived in the remaining 11 cases (interval = 8). Opacities with irregular or stellate margins were recorded in most cases (88%) at diagnosis. All cancers were invasive and larger than those in other categories.

Minimal signs were observed on the previous screening mammogram in 35 cases, mostly (31 of 35) in screen-detected cancers. Isolated microcalcifications were more frequently recorded at diagnosis compared with other categories (34% *vs* 18%), and pT and pN distribution was moderately less favourable than in mammographically occult cases.

At review of the previous screening mammogram, no mammographic abnormality was found at the cancer site in 73 cases classified as 'mammographically occult'. The masking effect of radiologically dense parenchyma may account for some cases, but a 'fatty' parenchymal pattern (N1–P1)

**Table II** Distribution of cases according to the review of previous screening mammograms and to other studied variables

| | | *Classification of previous screening mammogram* | | | |
|---|---|---|---|---|---|
| | *Total cases (100%)* | *Screening error (%)* | *Minimal signs present (%)* | *Occult (%)* | *Occult at diagnosis (%)* |
| Total cases | 134 | 16 (12) | 35 (26) | 73 (54) | 10 (7) |
| Age (years) | | | | | |
| 40–49 | 27 | 3 (11) | 6 (22) | 13 (48) | 5 (19) |
| 50–59 | 42 | 6 (14) | 12 (29) | 21 (50) | 3 (7) |
| 60–69 | 65 | 7 (11) | 17 (26) | 39 (60) | 2 (3) |
| Diagnostic modality | | | | | |
| Screen detected | 85 | 5 (6) | 31 (36) | 48 (56) | 1 (1) |
| Interval | 49 | 11 (22) | 4 (8) | 25 (51) | 9 (18) |
| Tumour appearance on the diagnostic mammogram[a] | | | | | |
| Opacity, sharp | 11 | 1 (9) | 4 (36) | 6 (54) | — |
| Opacity, undefined | 55 | 11 (20) | 13 (24) | 31 (56) | — |
| Opacity, stellate | 25 | 3 (12) | 6 (24) | 16 (64) | — |
| Calcifications | 28 | 1 (3) | 12 (43) | 15 (54) | — |
| Distortion | 2 | — | — | 2 (100) | — |
| Histological type | | | | | |
| Intraductal | 9 | — | 2 (22) | 7 (78) | — |
| Ductal invasive | 55 | 10 (18) | 16 (29) | 27 (49) | 2 (4) |
| Lobular invasive | 21 | 2 (9) | 5 (24) | 11 (52) | 3 (14) |
| Other | 49 | 4 (8) | 12 (24) | 28 (57) | 5 (10) |
| Tumour size at diagnosis[b] (mm) | | | | | |
| <10 | 33 | 2 (6) | 8 (24) | 23 (70) | — |
| 11–20 | 61 | 8 (13) | 17 (28) | 28 (46) | 8 (13) |
| >20 | 30 | 6 (20) | 8 (27) | 15 (50) | 1 (3) |
| Axillary nodes[c] | | | | | |
| Involved | 29 | 4 (13) | 10 (34) | 14 (48) | 1 (3) |
| Not involved | 93 | 11 (12) | 22 (24) | 52 (56) | 8 (9) |
| Wolfe parenchymal pattern of previous screening mammogram | | | | | |
| N1, P1 | 48 | 5 (10) | 10 (21) | 32 (67) | 1 (2) |
| P2, Dy | 86 | 11 (19) | 25 (29) | 41 (48) | 9 (10) |

[a]Diagnostic mammogram was not available in three interval cases. [b]Invasive cases only; not available for one interval case. [c]Invasive cases only; not determined for two cases.

was recorded in 44% of cases. Fast tumour growth may be another explanation for these cases, with cancers being under the threshold of detectability at previous screening. Nevertheless, pT (pTis–pT1a b = 41% *vs* 20%) and pN (pN0 = 79% *vs* 73%) distribution was particularly favourable with respect to other categories, whereas a less favourable stage distribution might be expected for fast-growing tumours.

No abnormality was observed either on the previous screening mammogram or on diagnosis in ten cases, mostly (90%) interval cancers. Invasive lobular histological subtype, which is known to be difficult to detect by mammography, was observed in three cases, whereas a 'dense', possibly masking, P2-Dy parenchymal pattern was observed in nine of ten cases.

### Discussion

Apart from the variability due to the limited sample size considered, the results of the present study were strikingly consistent with those reported by Van Dijck *et al.* (1993). Screening error or minimal signs of tumour were observed at the review of the previous screening mammograms in 31% of interval cancers, but also in 42% of screen-detected cancers, the latter figure being accounted for mostly by cases in the 'minimal signs' category.

Screening errors might be reduced by improving the quality of mammographic performance (especially as far as positioning is concerned) and by reducing the chance of misperception (e.g. double reading) but, according to our results, this would achieve earlier diagnosis in at most 22% of interval and 6% of screen-detected cancers.

Earlier detection of cancers showing minimal signs of tumour at the review of the previous screening mammogram would be much more promising, as 26% of all cancers were classified in this category. Such a goal might be achieved by adopting less stringent criteria for referral to diagnostic assessment, especially for opacities with undefined margins and isolated microcalcifications. Such a policy is just the opposite to that currently adopted, aimed at improving screening specificity, as shown by the low referral rate in both the Florence and the Nijmegen Programmes (Ciatto *et al.*, 1990). At the repeat screening round in our programme in the year 1992 the recall rate to assessment was 1.8%, the benign biopsy rate was 0.07%, the benign/malignant biopsy ratio was 0.13:1, the cancer detection rate was 0.51% and the prevalence of invasive cancers <1 cm was 0.22% (0.24% including pTIS). All these indicators suggest a high performance, comparable to other European programmes (Wald *et al.*, 1993), and well within the range of the recommended European standards (Kirkpatrick *et al.*, 1992). The majority of cases which could be detected earlier by a more aggressive diagnostic approach are in the 'minimal signs present' category, that is they have a benign mammographic appearance. Accepting less specific, benign mammographic signs as positive would considerably increase referral and biopsy rates, which might turn out to be unacceptable, as suggested by Moskowitz (1983).

Reducing the rescreening interval to 1 year could be proposed to decrease the interval cancer rate and to advance the time of detection of some screen-detected cancers. However, this would not be the case for interval cancers occurring in the first year of the rescreening interval (22 of 49 in the present study), or for interval or screen-detected cases which were radiologically occult at diagnosis. As suggested by Van Dijck *et al.* (1993), most screening errors would be diagnosed at screening 1 year later, as well as an unknown proportion of interval cancers of the second year and of screen-detected cancers in the 'minimal signs present' and 'radiographically occult' categories. In these cases the advance in detection time with respect to biennial screening would be at most 1 year, and it is questionable whether this would have any impact on further mortality reduction by screening, especially considering the favourable stage distribution presently observed in these subgroups (pTIS or invasive <1 cm = 77%, pN0 = 75%). Moreover, reducing the rescreening interval to 1 year almost doubles the cost compared with biennial screening, and the benefit would certainly be inferior to that obtained by investing equivalent resources to offer biennial screening to more women. Although we agree that a careful analysis of the cost-effectiveness of intensifying screening frequency is worthwhile, such a policy should be discussed only when the whole female population over 50 years old is covered by a biennial screening programme, which presently is not the case for the majority of European countries.

### References

CIATTO S, CECCHINI S, ROSSELLI DEL TURCO M, GRAZZINI G, IOSSA A AND BARTOLI D. (1990). Referral policy and positive predictive value of call for surgical biopsy in the Florence breast cancer screening program. *J. Clin. Epidemiol.*, **43**, 419–423.

FRISELL J, EKLUND G, HELLSTROM L AND SOMELL A. (1987). Analysis of interval breast carcinomas in a randomized screening trial in Stockholm. *Breast Cancer Res. Treat.*, **9**, 219–225.

GEDDES M, AMOROSI A, BALZI D, BIGGERI A, CAVALLINI V, CHELLINI E AND QUINN M. (1991). Tuscany Cancer Registry – cancer incidence and mortality in the province of Florence, 1985–1987. *Quaderni di Oncologia*, Vol. 4. Lega Italiana per la Lotta contro i Tumori: Florence.

KIRKPATRICK A, TORNBERG S AND THISSEN M. (1992). *The European Guidelines for Quality Assurance in Mammographic Screening*. Europe Against Cancer Programme: European Community, Brussels.

MARTIN JE, MOSKOWITZ M AND MILBRATH JR. (1979). Breast cancer missed by mammography. *AJR*, **132**, 737–739.

MOSKOWITZ M. (1983). The predictive value of certain mammographic signs in screening for breast cancer. *Cancer*, **51**, 1007–1011.

PACI E, CIATTO S, BUIATTI E, CECCHINI S, PALLI D AND ROSSELLI DEL TURCO M. (1990). Early indicators of efficacy of breast cancer screening programmes. Results of the Florence District Programme. *Int. J. Cancer*, **46**, 198–202.

PALLI D, ROSSELLI DEL TURCO M, BUIATTI E, CARLI S, CIATTO S, TOSCANI L AND MALTONI G. (1986). A case-control study of the efficacy of a non randomized breast cancer screening program in Florence, Italy. *Int. J. Cancer*, **38**, 501–504.

PEETERS PHM, VERBEEK ALM, HENDRIKS JHCL, HOLLAND R, MRAVUNAC M AND VOOIJS GP. (1989). The occurrence of interval cancer in the Nijmegen screening programme. *Br. J. Cancer*, **59**, 929–932.

VAN DIJCK JAAM, VERBEEK ALM, HENDRIKS JHCL AND HOLLAND R. (1993). The current detectability of breast cancer in a mammographic screening program. *Cancer*, **72**, 1933–1938.

VON ROSEN A, ERHARDT K, HELLSTROM L, SOMELL L AND AUER G. (1985). Assessment of malignancy potential in so-called interval mammary carcinomas. *Breast Cancer Res. Treat.*, **6**, 221–227.

WALD N, CHAMBERLAIN J AND HACKSHAW A. (1993). Report of the European Society of Mastology Breast Cancer Screening Evaluation Committee. *Breast*, **2**, 209–216.